# The Discovery of the Vital Principle, or the Physiology of Man

**Published:** 1839-04-01

**Authors:** 


### The Discovery of the Vital Principle; or the Physiology of Man. 8vo. pp. 566. London, 1838.

The present century will certainly be distinguished in the annals of the human mind. Every science and every art has received in it an impulsion which is rapidly hurrying it forward—the elements of political and social life have been analysed, and are in process of recombination—and, finally, the vital principle, the grand secret, is discovered.

Genius is proverbially modest. If a man invents a new tooth-powder, nay, if he gives only a new designation to an old one, he sets his name to the thing forthwith, and affects to consider immortality certain. But the discoverer of the vital principle is nameless. We see him not in the title-page, we seek him in vain in the preface, and we abandon in despair the search for his whereabouts. The discovery, however, will itself be a reward, more substantial and more glorious than the beggarly fame that waits on the breath of the million—a fame that is prostituted for the panders to their pleasures, or the destroyers of their kind.

It would be a waste of time to dwell on the importance of a knowledge of the Vital Principle. Common sense proclaims it. But it seems that that knowledge is peculiarly necessary now. Horrors are hanging over us, and there is something awfully significant in these hints:—

"The period, however, has arrived in which this truth must be developed! Real knowledge always progresses with necessity, and the elucidation of the vital principle has now become essential to the wants of mankind; for, while we continue, unacquainted with the origin of life, what effectual step can be taken to prevent the incursions of disease and death, so unusually prevalent in the present day? On the explanation of the vital phenomena, and general diffusion of physiological knowledge, will be found to depend the preservation of the entire human species." *Pref.* vi.

If this does not awaken attention we know not what will rouse people. We have been sleeping on a volcano. How grateful we should be to the gentleman before us. Would that we could call him by his name.

Perhaps there are few terms more used in medicine than "vital principle," "vital action," "vital laws." If there is a difficulty, they give us the solution—if a thing is incomprehensible, the laws of vitality explain it. The vital principle is, like Cerberus, "three gentlemen at once," and does all sorts of work for all sorts of people.

It may be doing a service to medicine, to lay before the profession a few of the leading notions on the nature of vitality. Those who make liberal use of the term, would be puzzled, perhaps, were they put in a corner, and asked to state exactly what they meant. Before, then, we announce the discovery of the genuine vital principle, the real Simon Pure, we will introduce a few of the sham ones.

1. *John Hunter's idea of Life.*—It is generally supposed that Hunter's vital principle is, like the "delicate Ariel," something very spiritual indeed. There cannot be a greater mistake, as the following passage from his work on the blood, which supplies us with his views in detail, will shew.

"This living principle in the blood, which I have endeavoured to show to be similar in its effects to the living principle in the solids, owes its existence to the same matter which belongs to the other, and is the materia vitæ diffusa, of which every part of an animal has its portion:* it is, as it were, diffused through the whole solids and fluids, making a necessary constituent part of them, and forming with them a perfect whole; giving to both the power of preservation, and the susceptibility of impression, and, from their construction, giving them consequent reciprocal action. This is the matter which principally composes

* "I consider that something similar to the materials of the brain is diffused through the body, and even contained in the blood; between this and the brain a communication is kept up by the nerves. I have, therefore, adopted terms explanatory of this theory; calling the brain the materia vitæ coacervata; the nerves the chordæ internunciæ; and that diffused through the body, the materia vitæ diffusa."

the brain; and where there is a brain there must necessarily be parts to connect it with the rest of the body, which are the nerves; and as the use of the nerves is to continue, and therefore convey, the impression or action of the one to the other, these parts of communication must necessarily be of the same matter; for any other matter could not continue the same action.

From this it may be understood that nothing material is conveyed from the brain by the nerves, nor, *vice versâ*, from the body to the brain; for if that was exactly the case, it would not be necessary for the nerves to be of the same materials with the brain: but as we find the nerves of the same materials, it is a presumptive proof that they only continue the same action which they receive at either end.

The blood has as much the materia vitæ as the solids, which keeps up that harmony between them; and as every part endued with this principle has a sympathetic affection upon simple contact, so as to affect each other, (which I have called contiguous sympathy,) so the blood and the body are capable of affecting and being affected by each other; which accounts for that reciprocal influence which each has on the other. The blood being evidently composed of the same materials with the body, being endued with the same living powers, but from its unsettled state having no communication with the brain, is one of the strongest proofs of the materia vitæ making part of the composition of the body, independently of the nerves, and is similar in this respect to those inferior orders of animals that have no nerves, where every other principle of the animal is diffused through the whole. This opinion cannot be proved by experiment; but I think daily experience shows us that the living principle in the body acts exactly upon the same principle with the brain. Every part of the body is susceptible of impression, and the materia vitæ of every part is thrown into action, which, if continued to the brain, produces sensation; but it (the materia vitæ) may only be such as to throw the part impressed into such actions as it is capable of, according to the kind of impression; so does the brain or mind. The body loses impression by habit, so does the brain; it continues action from habit, so does the brain. The body, or parts of the body, have a recollection of former impressions when impressed anew; so has the brain: but they have not spontaneous memory as the brain has, because the brain is a complete whole of itself, and therefore its actions are complete in themselves. The materia vitæ of the body being diffused, makes part of the body in which it exists, and acts for this part, and probably for this part alone. The whole, taken together, hardly makes a whole, so as to constitute what might be called an organ, the action of which is always for some other purpose than itself: but this is not the case with the brain. The brain is a mass of this matter, not diffused through anything for the purpose of that thing, but constituting an organ in itself, the actions of which are for other purposes, viz. receiving by means of the nerves, the vast variety of actions in the diffused materia vitæ which arise from impression and habit, combining these, and distinguishing from what part they come The whole of these actions form the mind, and, according to the result, react so as to impress more or less of the materia vitæ of the body in return, producing in such parts consequent actions. The brain, then depends upon the body for its impression, which is sensation, and the consequent action is that of the mind; and the body depends upon the consequence of this intelligence, or effect of this mind, called the will, to impress it to action; but such (sensation and action) are not spent upon itself, but are for other purposes, and called voluntary."

John Bell sarcastically remarked that "a mouthful of nonsense in latin" sounds infinitely better, than in the mother tongue. The "diffused *matter of life*" of John Hunter, looks awkward in English. But what shall we say when we are told that it is something like brain? We may imitate Polonius'

polite acquiescence with Hamlet, when the prince told him that the cloud was "like a camel"—"It *is* backed like a camel."

We should perhaps admire Hunter's notions more, if they possessed the merit of intelligibility. As they stand they seem, to vulgar eyes, to be inconsistent where comprehensible, and rather rhapsodical where incomprehensible, that is, in about four-fifths of their extent.

Hunter, for example, says that the living principle "principally composes the brain," and that the nerves are necessarily of the same matter. But as the blood has the living principle, that is a strong proof that the materia vitæ makes part of the composition of the body, independently of the brain and nerves. This appears very odd on the part of the living principle, for, if the brain and nerves are principally composed of it, there seems no necessity for its additional dissemination. Mr. Hunter, too, informs us that the living principle in the body acts on exactly the same principle as the brain. So, here is a principle which makes a great part of a particular organ, and which seems identified, in Hunter's idea, both with the *composition* and the *action* of that organ, operating just like another principle, universally diffused yet in no other place associated with such visible organization. This is undoubtedly very beautiful, but rather mysterious. The account of the diffusion of the materia vitæ is a fine piece of *chiaro-scuro*. Few exceed Hunter in this sort of thing. The passage is like the speech in Don Juan:—

"A good sample, on the whole,
Of rhetoric, by the learn'd call'd rigmarole."

Our poor faculties are left, we confess, at a humble distance, in this excursion of Hunter's genius. We are ashamed to say, that his meaning and his logic are equally beyond us.

Yet one thing does seem clear. If words go for any thing, Hunter tells us that the materia vitæ diffusa is "the matter which principally composes the brain." The whole gist of his argument, if argument such a rhapsody can be termed, goes to prove the analogy between the living principle and the brain. If this is not materialism, what is? Fortunately the bane is rapidly succeeded by the antidote, and the very next paragraph knocks the materia vitæ on the head.

"But mere composition of matter does not give life; for the dead body has all the composition it ever had. Life is a property we do not understand; we can only see the necessary leading steps towards it."

It is a pity that Mr. Hunter did not start with this. Had he only placed it before his theory of the materia vitæ, instead of after it, much trouble would have been spared to himself and to his readers. It contradicts point blank all that went before it. This mode of reasoning, though convenient in some respects, is open to some objection. It is difficult to know exactly what the author means, when he makes two assertions opposed to one another; and when he first explains a thing and then says it is inexplicable, we are tempted to inquire why he went to the pains of explaining it.

Mr. Hunter, however, has not done yet. The last paragraph left him asserting positively that mere composition of matter does not give life. In another place he goes even farther than this:—"Organization and life," he says, "do not depend *the least* on each other." This is pretty well—

vitalism run mad. But he sticks to it, for he declares that "organization and life are two different things." The materia vitæ diffusa is left in the lurch altogether, and no one would dream, from these expressions, that Mr. Hunter could have fathered that grown material bantling.

But, alas! this spiritualism was not deeply rooted in the mind of Hunter, and another material theory, different indeed from the "diffused" one, has issued from his physiological loins.

"Life, then, appears to be something superadded to this peculiar modification of matter: *or* this modification of matter is so arranged, that the principle of life arises out of the arrangement; and this peculiar disposition of parts may be destroyed, and still the modification from which it is called animal matter remain the same. If the latter be the true explanation, this arrangement of parts on which life should depend, would not be that position of parts necessary to the formation of a whole part, or an organ, for that is probably a mechanical, or at least an organical arrangement; but just a peculiar arrangement of the most simple particles, giving rise to a principle of preservation.... the arrangement for preservation, which is life, becomes the principle of action, not the power of action.... We have hitherto traced animal matter from its change from common matter to animal matter, the particles of which have possessed such an arrangement as to produce life."

Here life depends on the arrangement of the molecules. This is the common material doctrine. We shall shew, by-and-by, its fallacy; but it is evident that Hunter entertained it.

Neither vitalists nor materialists can appeal to John Hunter, or rather they may both do so. He has expressed all opinions, pleaded in a sorry fashion for all, and proved none. As an *argument* his reasoning is scarcely worth examination,—it is illogical, inconsistent, and hopelessly obscure; and his three contradictory assertions, each unqualified, do not leave him the merit of having discovered or announced truth, although he was unable to establish it.

*Mr. Lawrence's Theory of Life.*—This witty surgeon and able man, had a keen relish for the weaknesses of his predecessors or his peers, and he seldom scrupled to shew them up.

"The striking differences," so he spoke in the Theatre of the College of Surgeons, "between living and inorganic bodies, and the strong contrasts of their respective properties, naturally excited curiosity respecting the causes of this diversity, and endeavours to shew the mode in which it was effected. Here we quit the path of observation, and wander into the regions of imagination and conjecture. It is the poetic ground of physiology; but the union is unnatural, and, like other unnatural unions, unproductive. The fiction spoils the science, and the admixture of science is fatal to inspiration. The fictitious beings of poetry are generally interesting in themselves, and are brought forwards to answer some useful purpose; but the genii and spirits of physiology are awkward and clumsy, and do nothing at last, which could not be accomplished just as well without them; they literally incumber us with their help.

For those, who think it impossible that the living organic structures should have vital properties without some extrinsic aid;—although they require no such assistance for the equally wonderful affinities of chemistry, for gravity, elasticity, or the other properties of matter, a great variety of explanations, suited to all tastes and comprehensions, has been provided.

Some are contented with stating that the properties of life arise from a vital principle. This explanation has the merit of simplicity, whatever we may think of its profoundness: and it has the advantage of being transferable and equally applicable to any other subject. Some hold that an immaterial principle, and others, that a material, but invisible and very subtle agent, is superadded to the obvious structure of the body, and enables it to exhibit vital phenomena. The former explanation will be of use to those who are conversant with immaterial beings, and who understand how they are connected with, and act on, matter. But I know no description of persons likely to benefit by the latter. For subtle matter is still matter; and if this fine stuff can possess vital properties, surely they may reside in a fabric which differs only in being a little coarser."

Mr. Lawrence makes small bones of the vital principle. He is merry at the idea of its being immaterial, and almost as merry at the notion of its being something superadded to the organs, material in its nature, but more subtle than ordinary matter. These doctrines may be false, but we do not think them so ridiculous, as Mr. Lawrence would have them. Their absurdity hinges on their inconsistence with what we know. But do we know the properties of matter? do we know the constitution of the universe, so well as to erect that knowledge as the standard of unerring truth, and to measure the more recondite operations of Nature, by the acquaintance we possess with her coarser works? The force of the argument, and the justice of the sarcasm of Mr. Lawrence, hinge on the determination of this question; and perhaps in his wide acquaintance with biology, he has been enabled to determine it. For our own parts, we confess that our philosophy is less strong upon the wing, and while her feeble flights may have instructed us to doubt, they have not given us confidence to dogmatise. We are of the opinion of Horatio, and fancy there are "more things in Heaven and earth," than we have yet attained to dreaming on.

Fortunately Mr. Lawrence does not limit his efforts to the demolition of the hypotheses of others. He imitates the Brahminical Cosmogonists, and, feeling that the world is insecurely poised on the back of an elephant, he puts that elephant upon a tortoise.

"To call life a property of organisation would be unmeaning:—it would be nonsense. The primary or elementary animal structures are endued with vital properties, their combinations compose the animal organs, in which, by means of the vital properties of the component elementary structures, the animal functions are carried on. The state of the animal, in which the continuance of these processes is evidenced by obvious external signs, is called life."

It is, no doubt, absurd to affirm that life is the result of organization, for life precedes the existence of organs. But how come the primary structures endowed with life? Here is the physiological tortoise. It carries the elephant tolerably well, but we see nothing to support *it*. If we can get the primary structures endowed with life, the rest is all smooth sailing. The difficulty is in the endowment. If we look at the germ, it is yet an amorphous mass. But it is alive, and from its simple substance are gradually evolving those proximate elements which form the materials of the tissues and the organs. To say that those elements are endowed with life, is to say no more, as a matter of fact, than what is obvious and palpable; and, as a theory of rational causation, to say nothing at all.

*Theory of Life of Tiedemann, Treviranus, and of the Modern German School.*—In the physiology of Tiedemann, the source and nature of the vital power are considered rather in detail. Our readers may naturally anticipate much from the industry and profundity of the German School. They will presently perceive to what the researches of this School have led.

Tiedemann first shews the opposition of vital and chemical laws—the peculiarity of composition of organic matters—and the necessity of a special force to produce them.

"The principal result," he goes on to remark, "of the comparisons made between the composition of organic bodies and those of inorganic bodies, which comparisons are founded on observations and researches in the chemical composition of these bodies hitherto pursued, is that the former have peculiar matters, which we call organic, for their basis. The changes of composition which take place in bodies endued with life, are not simply the effects of affinities similar to those observed in brute or inorganic bodies; they are the effects of affinities and forces of a special nature. Organic matters are the only ones which exhibit, and for the greater period of time in a particular state of aggregation and form called organization, the manifestations of activity which we designate by the name of life. They are accumulated at one time in bodies actually living, and life is manifested in them; at other times they are exterior to living bodies, mixed with inorganic matters, and then only capable of living. In this latter state they may return to the domain of living bodies, and into the tide of life, either in the shape of aliments, or, in a direct manner, by the aid of certain circumstances, as happens in spontaneous generation. Purely chemical affinities, or the action of simple chemical forces, appear, in the present state of our planet, to produce no organic combination of matter, such as albumen, gelatin, starch, gluten, &c.; at least we possess no facts which go to support the contrary opinion. None but organic bodies themselves are capable of introducing inorganic matters into organic combinations, of which respiration in particular and the nutrition of plants are examples."

This is a sufficiently distinct admission of the existence of a special something, call it by what name we choose. It signifies little whether that name be vital principle, organic force, or any other designation—whether we suppose it immaterial, or subtly material. It is of a special nature, and so far from being the result of organism, its operation is necessary for the constitution and existence of organic matter. But M. Tiedemann proceeds:—

"If we extend our researches still further, a question presents itself, namely, how organic matters, their different combinations and living bodies, are formed in our planet? The solution of this problem passes the limit of our experience. Should we, however, wish to hazard an answer to it, we fall into the waste of conjecture, and are forced to erect hypotheses, which are but probable, and not at all certain. We suppose that organized bodies have existed in our planet from its commencement; or else we admit that organic matters and living bodies have been produced, under certain circumstances, together with the elements and inorganic matters, by the action of general physical causes; or, lastly, we conjecture that the substance of living bodies was primitively contained in water, as primitive organic matter, having the property of taking on the organic form, that it gave origin to organic bodies of very simple and varied kinds in consequence of circumstances, and that these bodies have passed successively to more complicated forms, until at length the generative organs and their manifestations of activity having appeared in them, they were endowed with the faculty of preserving themselves in a continuous manner, by means of generation, as separate species."

There is one hypothesis, that has at least some faint probabilities upon its side, to which M. Tiedemann does not even deem it necessary to allude —the hypothesis which accounts for the existence of animals by the will of a Creator. The modern Pantheists scout the idea. They see nothing but absurdity in the supposition of a Great First Cause, they deem the belief in a Deity as unfit to be even entertained by their philosophy, and they substitute in his place that most extravagant of all superstitions, that most grovelling of all religions—the self-created, self-endowed, and self-creating powers of Nature. The attributes of a Deity are reconcileable with common experience, and consistent with our ordinary knowledge of events. But this Pantheistical jargon is opposed in its essential features to all experience, and, while it professes to be the rational offspring of scepticism, it is really an acephalous monster, got from a heated imagination by credulity.

But let Pantheism speak for herself. Far be it from us to gag her mouth, and, if her cause is good, and her arguments are strong, if Truth be on her side and Reason her champion, Prejudice however lusty, Error however consecrated by the respect of ages, will ultimately quail before her.

" Geology," proceeds Tiedemann, " is opposed to the first hypothesis of the existence, in our planet, of living bodies from the first moments of its creation. Fossils are found only in the exterior crust, that is to say, in the superficial layers of the earth, the formation which is most recent, whilst there are none at all in the primitive earths. Consequently there was a time when no living being existed on the globe. Even supposing we admitted this hypothesis, we should still leave untouched the question, how living bodies were formed, inasmuch as we could say nothing concerning the mode of origin of our planet and of the bodies which constitute it. In reference to this question, it matters little whether we declare for vulcanism or neptunism, since the geologists are under the necessity of leaving the origin of fire and water without explanation, and the biologist is still less able to pronounce any opinion on that of living bodies.

The difficulties which occur in the second hypothesis, of the dependence of the production of organic matters and living bodies on the action of general physical forces, are, that we are actually in want of facts which would authorize us to conclude analogically that organic matters and living bodies can proceed from inorganic matters, never having observed any thing similar, at least up to this day. Far from this being the case, living bodies are unable to produce, with inorganic substances, the greater number of the materials which enter into their composition, and for such end they require the matter of other organic bodies, which they introduce into themselves. Plants are nourished principally by the remains of dead vegetables or animals: animals likewise preserve their existence by means of vegetables, and even of other animals.

The most probable hypothesis is the third, viz., that the substance of organic bodies existed primitively in water, as matter of a particular kind, and that it was there endowed with the plastic faculty, that is to say, with the power of acquiring, by degrees, different simple forms of living bodies, with the concurrence of the general influences of light, heat, and perhaps also of electricity, &c. and of then passing from the simple forms to other more complicated, varying in proportion to the modification occurring in the external influences until the point when each species acquired duration by the production and manifestation of activity of the genital organs. Although we cannot here also answer the question, whence came the water and the organic matter which it contained, yet this hypothesis is the one which accords best with the facts with which geology has latterly been enriched. In fact, we find no organized bodies belonging to what is called the primitive world in the strata of earths which modern geologists consider as the products of fire or of vulcanism. They are only observed in the upper layers of the earth, in those of the latest formation, and in the soils which have evidently been precipitated in the midst of the waters. Aquatic animals existed before terrestrial animals. An argument which favours the hypothesis according to which the organic kingdom has been gradually developed and elevated from simple to more complicated forms, is drawn from the fact that we meet with remains of organic bodies belonging to the most simple species in the secondary and more ancient soils, whilst the most recent strata of the earth contain the remains of more complicated living bodies. The soils which rest directly on primitive rocks, present fragments of corals, radiated animals, and shells. It is only after these that remains of vertebrated animals, fishes, reptiles, and cetacea are found in the water. Fossil bones of oviparous animals exist in the deep strata of the earth, whereas the viviparous mammiferæ are met with in the superficial layers. We observe the same in the organic complication of vegetables, whose remains are contained in the different layers of the earth. Impressions of cotyledonous plants, especially of ferns, are the first vegetable traces met with in the deep-seated strata. Then come the remains of monocotyledonous plants, of aborescent gramina, of palms, &c. and finally those of the coniferæ and other dicotyledonous plants.

There have not yet been found any fossils belonging to apes or man, whose organization has reached the highest degree of complication and development. We may therefore admit, with great probability, that apes and men are the last and the newest products of our planet."

We have allowed Pantheism to plead her cause, and the issue is now before the tribunal of science. If she is credible, organic matter diffused in water had inherently a plastic power, by which it developed itself from simpler forms to higher, until the generative organs were obtained, and the forms then reached were ever after perpetuated!

We say nothing of the absence of proof of any plastic power of the sort. That must be taken for granted. But granting it, when and why did such power cease? If it existed at all, it was inherent—if it was inherent, it must exist still—if it still exists it must be capable of observation, of examination, of demonstration. But who ever observes, examined, or can demonstrate anything approaching to it?

The Pantheist may reply:—" This plastic power, in which I demand a blind belief, worked till it produced the generative organs and then ceased. It had accomplished its mission, or rather had effected its intention, and though I laugh at the idea of intelligence in your Deity, you must assent to its operation in mine. My plastic power of organic matter first worked that matter into a zoophyte. From the zoophyte it developed itself into an articulated animal. It next grew a mollusk—a fish—a reptile—a bird—a mammal—and the hydra viridis was but the inceptive of a man—a man the completion of a hydra viridis. Through each successive step my plastic power went, and, when it got to man it stopped. Suddenly all the creatures below him, and at the same instant himself, were provided with genital organs, and a pair of each creatures was at once transformed by the magic wand of my plastic power into male and female."

Such is the ingenious theory of Pantheism. We ask not *how* these genital organs, which were to extinguish the plastic power came to be its "crowning mercy." But for the hypothesis to be correct, it is obvious that the earlier creatures *must have been unprovided with them.* If the earlier races had them, that ought from the very terms of the theory to have put a stop to the plastic business. Have the Pantheists proved that they had them not? Do they attempt to prove it? Are they not conscious that the very notion has only to be promulgated to cover them with ridicule? There is no proof, no probability, not the shadow of a shade of likelihood that the very earliest creatures who sprang into existence in the infant world, did not perpetuate their like, as their descendants do, and gradually stocked the waters with their offspring, just as any modern zoophyte, or mollusk may.

Such is the baseless fabric of the supposition (we can hardly dignify it by the name of scientific theory) that a plastic power operated, till it effected organs of generation, and then nobly committed felo-de-se. But there is another term in the proposition, another condition of its truth, which we may as well dissect, and expose the real nature of. For that proposition to be true, it is not only requisite that the generative apparatus should have lain in abeyance until the present scheme was arrived at, but it is also requisite that the animals which existed in the younger world should be *inferior in organization, and less perfect in development, than those which are found at present.* Is this the case? Is the animal icognitum, is the mammoth, are the mollusca instances of such a fact? They are notoriously the contrary. They are as perfect samples of their class as any mollusk, reptile, or mammal of our day, and to suppose that these are developed from those, is to fly in the face of consistency and science.

We need not pursue this childish folly farther. It is opposed to all experience, to probability, to reason; and the man who can reject the simple doctrine of a God and a creation, for this godless, self-existing, self-destroying, self-contradicting, senseless, aimless crotchet, must be the most credulous slave to superstition that ever formed the raw materials of a lunatic.

Contrast it with the creed of a believer in the Deity :—*that* the world in its infancy presented important differences of condition from its present—*that* it was stocked by the all-wise Creator with animals adapted to its state—*that* one, perhaps more than one, revolution occurred in its constituent parts —*that* with the change of circumstances in their situation, the same Creator willed new beings to inhabit it—and *that*, so far as our knowledge reaches, the earlier creatures and the old, had similar laws of organization and of reproduction.

We ask, and we ask with confidence, which doctrine—Theism or Pantheism—draws least upon our faith, squares best with what is known—which doctrine demands most abasement of reason, breeds most superstition? For ourselves we do not scruple to pity the Pantheist, as the most credulous of Pagans, the most abject worshipper of effects—the visible phenomena of Nature.

It has been supposed that minute anatomical knowledge, and that deep physiological researches incapacitate for general ideas of magnitude, and, as in the analogous case of law, confine and cripple the mind to precedents, and to the close and foggy atmosphere of what is demonstrable immediately around it.

The samples we have offered might countenance the supposition. Were it true, anatomy and physiology would be indeed degraded. But it is not true.

The progress of Science dispels the errors which herself created, and if Hunter, and Lawrence, and Tiedemann have advanced absurd or pernicious doctrines, the discoveries to which their examinations led, have brought a sufficient antidote. There are some who are alarmed at the advance of knowledge. But truth has nothing to fear from that, and none can contend that error *ought* to be perpetuated. In the present case it is imperfect knowledge that is formidable. With the growth of genuine information, we find the arrogance and dogmatism of an earlier epoch vanish, and the better we understand the operations of Nature, the more clearly we perceive the presiding hand of Intelligence. To prove what we have said, we shall quote the opinions of Müller, one of the last and of the best of physiologists, one on whose page the present state of physiology is written.

"Some have believed," he says, "that life,—the active phenomena of organised bodies,—is only the result of the harmony of the different parts—of the mutual action, as it were, of the wheels of the machine,—and that death is the consequence of a disturbance of this harmony. This reciprocal action of parts on each other, evidently exists; for respiration in the lungs is the cause of the activity of the heart, and the motion of the heart at every moment sends blood, prepared by respiration, to the brain, which thus acquires the power of animating all other organs, and again gives occasion to the respiratory movements. The external impulse to the whole machinery is the atmospheric air in respiration. Any injury to one of the principal moving powers in the mechanism, every considerable lesion of the lungs, heart, or brain, may be the cause of death; hence these organs have been named the *atria mortis*. But the harmonious action of the essential parts of the individual subsists only by the influence of a force, the operation of which is extended to all parts of the body, and which does not depend on any single part, this force exists before the harmonizing parts, which are, in fact, formed by it during the development of the embryo. A complicated piece of machinery, constructed in adaptation to an end,—for example, a watch,—may present an action resulting from the co-operation of individual parts, and originating in one cause: but organic beings do not merely subsist by virtue of an accidental combination of elements; the vital force inherent in them itself generates from organic matter the essential organs which constitute the whole being. This rational creative force is exerted in every animal strictly in accordance with what the nature of each requires."

Müller goes on to observe:—

"The unity resulting from the combination of the organising force with organic matter could be better conceived, if it could be proved that the organising force and the phenomena of life are the result, manifestation, or property of a certain combination of elements. The difference of animate and inanimate organic matter would then consist, in that state of combination of the elements, which is necessary to life, having in the latter undergone some change. Reil has stated this bold theory in his famous treatise on the 'vital energy,' which some physiologists,—Rudolphi, for example, regard as a masterpiece, on which the principles of physiology must be founded.

Reil refers the organic phenomena to original difference in the elementary combination and form of the organic bodies. Differences in the mode of combination of the elements and in form, are, according to his theory, the cause of all the variety in organised bodies, and in their endowments. But if these two principles be admitted, still the problem remains unsolved; it may still be asked, how the elementary combination acquired its form, and how the form acquired its elementary combination. That the form of the organic matter does not determine originally the mode of its action, is proved indisputably by the the fact, that the matter from which all animal forms are produced is at first almost without form. The germ in all vertebrata, and probably also in the invertebrata, from what is known of a few species, and from what I have observed in the planaria, is a round disk of simple matter; here is no difference of form corresponding to the difference of the animals. On the other hand, the form of inorganic bodies is always determined by their elements, or by the combination of their elements. And this Reil himself admits; for he says: 'Form of matter is itself a phenomenon, which depends on another phenomenon, namely, the elective affinity of the elements and their products.' Hence it would follow, that if the elementary combination were alone the cause of the organic forces, this elementary combination itself would be at the same time the formative principle. Now, since in organised bodies immediately after death, the elementary combination does not appear to be different from that of bodies still living, Reil must admit the existence of other more subtile matters not recognisable by chemical analysis, which are present in the living body, but are wanting after death. Into the composition of the organic matter of the living body there must enter an unknown (according to Reil's theory, subtile material) principle, or the organic matter must maintain its properties by the operation of some unknown forces. Whether this principle is to be regarded as an imponderable matter, or as a force, or energy, is just as uncertain as the same question is in reference to several important phenomena in physics; physiology in this case is not behind the other natural sciences, for the properties of this principle in the functions of the nerves are nearly as well known as those of light, caloric, and electricity, in physics."

Our readers are probably aware, that the "proximate principles" of organization are combinations of three or of the four elements, oxygen, hydrogen, nitrogen, and carbon. But inorganic compounds are usually either binary, or combinations of binary arrangements. Were Reil's theory true, the peculiarity of organization would result merely from the fact of ternary or quaternary composition. But the general law of inorganic matter is binary or compound binary arrangement. How then, in the case of organic matter, is the usual arrangement altered. It is clear that there is a special force. It matters little what that force is called—"life,"—"anima"—"archæus." It differs from the law of inorganic matter, has properties and displays phenomena of its own, and those phenomena and properties are as legitimate grounds for observation and for reasoning, as the laws of light, of electricity, or gravity.

*The Discovery of the Vital Principle.*—If we have hitherto advanced with difficulty, uncertain where to tread, and scarcely able to distinguish truth from error, our hesitation will speedily be exchanged for confidence—our difficulties and doubts for the fulness of belief—our painful search for truth for the rich possession of her. The discoverer of the vital principle is before us, and that discovery, the monument of his genius or his fortune, is in actual print. The subtle essence that escaped the observation of Harvey, Haller, or Hunter, has been happily distinguished and boldly grasped by the "ουδεις" of the present page, and the 19th century has the honour of witnessing the publication of a secret, to which no others are fit to hold a candle.

The discoverer informs us, that:—

"The intention of the present work will be evident on the perusal of the following brief outline of its contents.

Animal life being divided into three distinct stages, and these proving analogous to the solid, fluid, and aeriform states of inorganic matter, all creation is here identified with the *animal origin*, and our universe, generally admitted to be in the condition of an undulating fluid, maintained to be capable of assuming also the solid and gaseous forms of existence: from which the following deductions have been made:—

1st, That an Ovum was originally formed, containing within its circumference or boundary, all known matter in a solid, latent, and inactive condition. From this ovum has arisen,

2dly. The Fœtal, or fluid state of matter, which has been endowed with active life for the purposes of organization; the heart being our sun, the other heavenly bodies—the several organs belonging to the fœtus: the whole (our earth included) progressing at the present period towards the structure of one complete human frame, analogous to that of *Man*.

3dly. The future, or perfected existence of the present fœtus as a locomotive being of celestial substance and imperishable nature: this last period answering to the life of man subsequent to birth, which is the aeriform stage."—*Pref.* vii.

We have often heard of the value of analogy. Its force in argument is happily exemplified in the preceding passage. Who would have dreamt that the universe was once an ovum? Who, if he had not been told that the present is the fœtal state of matter, would have perceived that wonderful truth? Who would have guessed that the future was "a locomotive being?" And yet a little consideration might have hinted it; for the future means what is to come, and coming implies locomotion.

The solid form of matter, the ovum of the universe is (listen reader) the diamond!

"The diamond is, in my opinion, the identical primitive matter so correctly defined by the poet and philosopher I have just quoted. At first view it may appear a strange and bold assertion, but I expect to prove, beyond a possibility of doubt, that from this material the universe, containing such an immense number and variety of bodies, was really organized. According to Pliny, 'the superior rigidity of the diamond renders it proof against almost every species of blow; insomuch, that if beaten on an anvil, the iron itself, both of the anvil and of the hammer, will yield before the diamond." 7.

Thus, we are all formed from diamonds. It has often been remarked that the germs of discovery have appeared among the vulgar. The Irish, that ingenious people, who notoriously have diamonds of their own,* naturally obtained a clue to the truth, and have infused their knowledge into their popular expressions. The force of "Och, my *jewel*, be aisy," must be apparent.

The ovum then was a diamond. It sprang into a fœtus.

"The fœtal life of this universe commenced with the first spark of electricity, elicited by friction in the centre of the ovum: on entering into this second stage of existence, primitive matter became changed from the oval to the cordiform shape, which transformation took place in the following manner.

Heat, with some few exceptions, is well known to ascend. In boiling a kettle of water (to use this very familiar illustration), the heat commences from the bottom (or source), and forces its way up to the top, forming in its ascent a line of levity, till it finally escapes and gravitates as steam. Now the particles thrown

* "Irish diamonds," unreasonably depreciated.

off from the ovum, in the first instance, must have been projected upwards from the boiling point of matter, forcing their ascent in proportion to the violence of the stroke by which they were propelled. Losing gradually their elasticity, or fluid heat, these atoms, in falling, must have described the shape of a heart, and all converged to the freezing point, which, we perceive, must be situated on the line of gravity of the ovum immediately underneath its apex, or coldest portion. This lower part of the ovum answers to the bottom of a kettle, which is always comparatively cool, while its top is in a boiling state. If the line I have described be drawn from this freezing point through the centre of the ovum to the opposite extreme or boiling point of its surface, it will cut the *poles* of our sun, and form the line of Apsides, which is the spine or gravitating line of the fœtus. This line extends to the extremities of the universal sphere, dividing the sun or heart into two cavities, similar to the structure of that of a human being. In the line of gravitation are situated the powers of galvanism (or animal magnetism), and electricity; both of which belong to animal life, and result from gravitation or pressure. Galvanism is elicited from fluid or boiling atoms, electricity from solid ones. Electricity, at the freezing point, awakens the latent spark of life; galvanism prescribes bounds to it; the central point on the line between these two forces of galvanism and electricity being our sun, the seat of life of the fœtus; life being, by these two opposite forces, kept active. The matter which becomes electric by means of heat, it is well known, is thus made to diverge: this species of matter identifies itself with that thrown off at the South Pole (or positive point of the universe), and conducts electricity: the law of repulsion by which such matter is caused to diverge, is situated in the point of galvanism; hence a spark is elicited and the matter diverges in the heart form. The non-conductors, on the other hand, require friction to produce electricity: such is the quality of matter in the negative or freezing point of the gravitating line, the North Pole of our universe, where is situated the force of attraction, by which it is made to unite and become electric; but no visible spark is elicited, though one is certainly the result of such union." 22.

How simple was the transformation into a fœtus, how clear all the subsequent changes! We take shame to ourselves for never having suspected them. Our author, it will be noticed, assumes nothing. Every step that he takes is sure, every assertion is proved. What can be more lucid than the passage we have quoted, what can be more convincing? The world *is* a fœtus. If evidence were wanting, it would be found in what follows:—

"The whole of this vast machine of the universe has progressed from one simple solid egg of diamond. This egg, the heart and centre of our sun, is in a constant state of combustion, throwing off from its mouth or cavity particles of boiling hot diamond, which liquid flame rises to a certain height by the combustion or electric force with which it is projected from the body of the sun, and then losing its heat, gravitates (as water does from a fountain) to his apex, which is naturally adapted for the deposition of planetary bodies. Here the matter becomes stationary, uniting by the attraction of cohesion into the primitive or oval form, and in this way have all the planets been first generated from the body of the sun in the order of their respective orbits, from Mercury outwards to the Georgium Sidus, each becoming in turn the extreme point of cold, (or apex of the universe,) and extending the sphere of the fœtus." 35.

A chapter on "matter" from such a hand may be well supposed to display peculiar excellences. It does; and, not the least, is the hearty freshness with which the author promulgates his notions. There is no trimming, no shuffling—the Discoverer of the Vital Principle feels that he has a right to the discovery of everything else, and he does discover many things with wonderfully little trouble. For example:—

"All matter composing this universe is diamond; but that material varies according to combustion. At the point of extreme heat it is in a perfectly elastic, pure, and simple state; at the freezing point it becomes condensed or crystallized into what we term *Iron*. Now, all enquirers into the origin of matter have, by common consent, traced it to hydrogen and oxygen. I shall go a step farther, and assert that diamond is the base of hydrogen: iron that of oxygen. Hydrogen being matter in the point of extreme heat, oxygen being the same material in its most condensed state at the freezing point. These two gases, when combined, constitute *water*, which is our gravitating element. We shall, accordingly, find that the particles of matter deposited by the sun at our planet are, in fact, composed of diamond or hydrogen, and iron or oxygen, accompanied always by a certain proportion of *carbon* (or diamond in a cinereal state), all which are to be traced to the simple diamond." 62.

If that is not philosophizing we know not what is. Our author has a great advantage. Having found out all the secrets of Nature, he is not obliged to resort to the clumsy manipulations of chemistry. He need not decompose, nor recompose. He asserts that diamond is iron, and iron oxygen. To prove it, all that he has had to do, has been to go "a step farther." All other proof would be superfluous.

We have said enough to introduce our readers to the vital principle. They will not probably anticipate it, but we are sure they will anticipate something racy. What do they think of *dew* being the vital principle?

"Dew, which is quietly descending to us, to cool the vapour of the globe, has not yet been sufficiently investigated. There are some seasons of the year during which life would be insupportable without these most refreshing heavenly drops. It is well known how much dew contributes to the growth of plants: who has not noticed that during the greatest heat of summer, when dew falls most abundantly, and rain frequently descends in torrents, that not only is vegetation most rapid in its growth, but animals and worms of various kinds, and insects of every description are generated? *Dew, or rain,* however, has never yet been considered the *primary* cause of animal and vegetable organization. From what I can collect from Dr. Wells and other authors, I feel convinced that dew does not belong to our globe, the earth, but that it comes directly from the sun to us, and is always accompanied in its descent with black specks, similar in appearance to soot or flakes of charcoal; these black flakes and specks, during the severity of our winter, become, with the dew and rain, crystallized, and fall to our earth as snow. Thus it will be seen that dew, when deposited upon our earth, is not a simple but a triple substance, viz., carbon, hydrogen, and oxygen. Who has not experienced the unpleasant sensation caused by snow after it has lain on the ground for a day or two? It no longer retains that beautiful and primitive whiteness which it had on its first descent to the earth: on the contrary, it becomes dirty and spotted, dark, cold, and comfortless, and uneven on its surface. This aspect of snow is not confined to towns or their vicinity, where it might be conjectured to arise from smoke and dust descending from our atmosphere: no, snow presents the same appearance on barren heaths, on mountain tops, or the wildest plains." 172.

Dew, then, plays first fiddle in the scheme of Nature.* How it becomes the vital principle is not, at first sight, clear. But, perhaps, the following considerations may enlighten us.

---

* This sufficiently explains the popularity of "Mountain Dew."

" Oxygen is the consolidation of the liquid hydrogen into metallic substance. Carbon is the cinder, or dust, resulting from the union of the two former. These elementary bodies are always united: they are all necessary to the existence of each other, and can never be entirely separated; when, however, these three materials come into close contact by surrounding pressure on the atmosphere, the electric spark on liquid flame escapes, and locomotive animal life is generated. In the aerial species of animal life, such as birds and insects of every description, particularly flies, the carbon and hydrogen predominate. In the larger animals that move on the solid body of the earth, the hydrogen and oxygen prevail. In the lowest species of the creation, such as burrow in the bowels of the earth, the carbon and the oxygen must always predominate; for however low and degraded the animal may be in the scale of life, still oxygen must be the base, and form the first germ of terrestrial animal structure, carbonic acid gas forming the fluid atmosphere surrounding every animal whether simple or compound in its mechanism." 183.

It would be superfluous to argue upon this. The vital principle is discovered. We may shout, like Xenophon's companions, " θαλασσα, θαλασσα!" Dew is at the bottom of it. Just conceive the simplicity, demonstrability of the theory. Theory do we call it—the truth—the axiom. People have been sleeping for forty centuries in the open air and never found out that dew, and nothing but dew, is the vital principle. Dew comes from the sun,—the sun is a diamond,—we are bits of diamonds, "chips of the old block"—of course dew *must* be the vital principle. The wonder is, not that dew is life, but—that we never found it out. Hail, nameless author! greater than Harvey, why wilt thou hide thy name?

Our readers must have been already struck with the magnificent conceptions of the discoverer of the vital principle. One who could find that principle in dew, would be pretty certain to make some other hits in physiology. In a chapter upon man, which ought undoubtedly to have been incorporated into a Bridgewater Treatise, and which may yet be amplified into one, we are gratified with the " ovum" of ideas, which, if so grand in their miniature condition, will indeed astound when developed into their " fœtal state."

There are seven organs, the exact number of the Seven Champions of Christendom, with which *we* do no doubt they have a close connexion.

" Each organ has its boiling and freezing point, or a line of gravity peculiar to itself, (see page 31 and 32 of this work,) and may be said to resemble a jette d'eau and filtering machine. While these organs filter through their sides or coat, they are also throwing off from their upper surface (or cavity) a continual stream of boiling fluid diamond, by means of the veins and arteries, which material descending to its gravitating point, (the point of extreme cold,) forms fresh provisions for the organs or vessels beneath. Wherever the veins and arteries terminate, the nerves commence. These last have their origin in the uterus, and terminate in the brain. The deposition of matter in the uterus occasions a boiling electric fluid to arise from thence, which, forming vessels in pairs, ascends to elongate the spine, and finally deposits at the extreme point of ascension the material which constitutes the brain." 209.

It would be useless to comment on these sublimities. As they pass the limit of ordinary understandings, and rise superior to the forms and substance of mere reasoning, the only thing left is to fall prostrate and adore in the Mussulman fashion. " Alla il alla, there is but one vital principle, and the great unknown is its prophet." We cannot resist the opportunity of giving a sketch of this huge philosopher's fœtal circulation.

"The heart, veins, arteries, and lungs are all filled with hydrogen, and the child comes into the world gasping for life with its mouth open." In this interesting situation, "the oxygen of the atmosphere coming in contact with the hydrogen, inflates the lungs, and, with the utmost velocity, gives an impetus to the expiring heart. It is in fact the contraction or condensation of the outward fluid amnios pressing the white blood (or hydrogen) back again into the cavity of the heart, which had rejected it, that causes an extraordinary effort in that organ to relieve itself, and, with one volcanic or muscular opposing effort, to throw the whole of its fluid contents to the outward surface. Thus is given an impetus to the whole muscular frame of the almost exhausted fœtus, while the fluid repelled to the surface, assists to throw the helpless foundling babe on the shore of an unknown island." For scientific truth, dramatic interest, simple grandeur, and domestic pathos, *this* fœtal circulation seems unequalled.

It might reasonably be supposed, that man being made of diamond, was likely to last. But there is one disturbing circumstance, which our readers have not taken into the account. He is a great worm manufacturer.

"Man is a complete manufactory of worms: every organ of his frame being built from them, and cemented together to form one grand whole. When, therefore, by any means, this cement is loosened by its elastic property becoming decomposed, the worms are set at liberty, and become themselves active living fœtuses; gradually making their way to the surface of man's body, they raise in his skin tubercles of various forms and sizes, like unto the hillocks and mountains upon the earth's surface, and, like them also, they burst forth and form volcanic eruptions, emitting their lava, by the attraction of which new depositions are formed from the external atmosphere." 468.

"Man, indeed, is (and I feel assured the theory cannot be denied,) formed of a complete tissue of worms. From the very centre of the system unto the surface, his whole body is nothing but a continuation of worms, all linked together to form one tree or rock, rising up perpendicularly to the earth's surface."

The worms themselves are made of diamond too, and there could not be a more happy illustration of the branch of art popularly denominated "diamond cut diamond," than this.

Divines are but too sensible of the consequences of the first great disobedience. We know not how they will receive the intelligence, that the forbidden fruit was—malic acid—and that it grew in the United Kingdom. Yet the unknown proves this as satisfactorily as he proves any thing.

The plot naturally thickens as we approach the termination of the volume. It seems that man, by the increase of his mechanical power, has formed new plans quite contradictory and inferior to the mighty plan of God: an error which has been creeping on ever since the flood, and has now become one frightful vice. Our situation is therefore very critical. The whole race of human beings, is on the very brink of being turned again into diamonds, and a strenuous effort is necessary on the part of each individual. We must make a long pull, a strong pull, and a pull altogether.

The remedy is obvious. Diffuse a knowledge of the vital principle. Make every man his own physiologist and doctor.

"Away, then, with every imaginary distinction of birth, of employment, or of country! we all are of one family—descended from one parent; our common country—the whole habitable globe; our employment—the one end and aim of perpetuating and improving the human species! Medical information must be extended without reserve to every branch of the community, in order that each individual, by the preservation of his own health, may contribute his share towards purifying the atmosphere." 542.

Can anything be more easy than that? We have been exhaling carbonic acid for sixty centuries. Let every man be his own doctor, and exhale nothing but oxygen.

As subordinate items of this glorious plan, we must warn our readers to prepare for emigration on a large scale. The unknown addresses to them this inspiriting address:—

"Make ready to set sail to the east—to the west—to the north—to the south! A discovery more brilliant than that of Columbus awaits you! a new, an unexplored region is about to be revealed—a country already the abode of your fathers, your mothers, your sisters, your lovers, and your friends!" 544.

For those who live inland, and cannot set sail direct from home, this man of wonders has a remedy. It is to construct Nassau balloons.

"The human mind, by this time, will comprehend, from the perusal of the preceding pages, how a remnant of the virtuous portion of mankind may be saved alive in the last day by the very powerful invention of balloons. Ere the awful termination of the present abode of man, let us hope we may see the surrounding atmosphere spotted and illumined by moving vessels of every description, as we now behold them on the waters. Here then is excitement for genius and talent to unite in every possible way; not a moment should be lost in devising the means of preservation from the impending danger." 393.

We are exhausted with the grandeur of these pages. We have done our duty. The readers of the Medico-Chirurgical Review, at least, will be saved, and all we ask is to order their booksellers in the New World to continue to take *us* in. Surely this is moderate and modest. An ordinary person might suppose that the discoverer of the vital principle had either just escaped from a keeper, or was in immediate want of one. A base reward for a man who has shewn us that our real composition is malic acid and diamond, and has told us of a certain method of cheating Death and the Devil.

